# Radiofrequency Currents Modulate Inflammatory Processes in Keratinocytes

**DOI:** 10.3390/ijms251910663

**Published:** 2024-10-03

**Authors:** Elena Toledano-Macías, María Antonia Martínez-Pascual, Almudena Cecilia-Matilla, Mariano Bermejo-Martínez, Alfonso Pérez-González, Rosa Cristina Jara, Silvia Sacristán, María Luisa Hernández-Bule

**Affiliations:** 1 Photobiology and Bioelectromagnetic Lab, Instituto Ramón y Cajal de Investigación Sanitaria (IRYCIS), Hospital Ramón y Cajal, Crta. Colmenar Viejo, km. 9.100, 28034 Madrid, Spain; elena.toledano@hrc.es (E.T.-M.); m.antonia.martinez@hrc.es (M.A.M.-P.); rosacristina.jara@salud.madrid.org (R.C.J.); 2 Angiology and Vascular Surgery Service, Hospital Ramón y Cajal, Crta. Colmenar Viejo, km. 9.100, 28034 Madrid, Spain; almudena.cecilia@salud.madrid.org (A.C.-M.); mariano.bermejo@salud.madrid.org (M.B.-M.); 3 Dermatology Service, Hospital Ramón y Cajal, Crta. Colmenar Viejo, km. 9.100, 28034 Madrid, Spain; pg.l.alfonso@gmail.com; 4 Aptamer Group, Histology Lab, Instituto Ramón y Cajal de Investigación Sanitaria (IRYCIS), Hospital Ramón y Cajal, Crta. Colmenar Viejo, km. 9.100, 28034 Madrid, Spain; silvia.sacristan@hrc.es

**Keywords:** keratinocyte, cytokine, NF-κB, MMPs, EGFR, ERK1/2, radiofrequency, physical therapies, inflammation

## Abstract

Keratinocytes play an essential role in the inflammatory phase of wound regeneration. In addition to migrating and proliferating for tissue regeneration, they produce a large amount of cytokines that modulate the inflammatory process. Previous studies have shown that subthermal treatment with radiofrequency (RF) currents used in capacitive resistive electric transfer (CRET) therapy promotes the proliferation of HaCat keratinocytes and modulates their cytokine production. Although physical therapies have been shown to have anti-inflammatory effects in a variety of experimental models and in patients, knowledge of the biological basis of these effects is still limited. The aim of this study was to investigate the effect of CRET on keratinocyte proliferation, cytokine production (IL-8, MCP-1, RANTES, IL-6, IL-11), TNF-α secretion, and the expression of MMP9, MMP1, NF-κB, ERK1/2, and EGFR. Human keratinocytes (HaCat) were treated with an intermittent 448 kHz electric current (CRET signal) in subthermal conditions and for different periods of time. Cell proliferation was analyzed by XTT assay, cytokine and TNF-α production by ELISA, NF-κB expression and activation by immunofluorescence, and MMP9, MMP1, ERK1/2, and EGF receptor expression and activation by immunoblot. Compared to a control, CRET increases keratinocyte proliferation, increases the transient release of MCP-1, TNF-α, and IL-6 while decreasing IL-8. In addition, it modifies the expression of MMPs and activates EGFR, NF-κB, and ERK1/2 proteins. Our results indicate that CRET reasonably modifies cytokine production through the EGF receptor and the ERK1/2/NF-κB pathway, ultimately modulating the inflammatory response of human keratinocytes.

## 1. Introduction

Inflammation is a highly complex physiological process that occurs in response to exogenous damage to the skin barrier or to internal deregulation. In the skin, inflammation occurs in response to wounds, trauma, or burns. This damage is often mild and therefore the inflammatory response generated and the repair process are self-limiting, preventing inflammation from persisting over time. When the inflammatory process is perpetuated over time, chronic inflammation is generated and inflammation ceases to be a physiological response and becomes a pathological situation. These chronic inflammatory processes can lead to degeneration of the affected tissues [1].

The use of anti-inflammatory therapies that restore skin balance is essential for the control of skin inflammation. Glucocorticoids have been the main tool available for decades; however, their inability to completely control chronic inflammation in most cases, as well as its medium and long-term adverse effects, has made the development of new treatments necessary [2]. Therapeutic strategies that inhibit the synthesis of pro-inflammatory cytokines represent a new modality of pharmacological therapy with significant clinical relevance due to their high therapeutic efficacy and specificity. However, allergic reactions and an acceleration of the elimination of antibodies against cytokines [3] have been reported. From this need, the development of new therapeutic strategies for chronic inflammatory processes has arisen.

Physical therapies are an alternative to conventional pharmacological treatments. These therapies include light-based therapies or those that apply electromagnetic fields or electric currents at different frequencies. Evidence suggests that intense pulsed light (IPL) [4] and a pulsed dye laser (PDL) [5] can effectively and safely improve acne vulgaris and rosacea. For other inflammatory skin diseases such as Darier’s disease [6], benign familial pemphigus (Hailey–Hailey disease) [6], psoriasis [7], atopic dermatitis [8], lichen planus [9], hidradenitis suppurativa [10], cutaneous lupus erythematosus [11], and cutaneous sarcoidosis [12], as well as multiple pigmentary disorders, these light-based therapies are an excellent complement to standard treatments, although their efficacy as monotherapy is often not sufficient to be used as sole therapy.

Light-based therapies, despite having an excellent safety profile, are not free from complications, including transient erythema, edema, pain, and burning sensation, as well as the possibility of hyperpigmentation or the formation of burns and blisters in the most severe cases [4]. These adverse effects sometimes make it necessary to discontinue treatment due to poor tolerance by patients.

A theranostic approach uses ionizing radiation through theranostic radiopharmaceuticals which combine diagnostic imaging and therapeutic intervention in a single procedure. These therapies are targeted, allowing treatments to be personalized, including to genetic and molecular profiles [13]. However, this technique involves complex procedures, high costs, and exposure to ionizing radiation, which, although generally low, can pose risks, especially with repeated treatments [14].

Radiofrequency (RF) offers several advantages over therapies that use ionizing radiation. RF is a noninvasive technique that uses electromagnetic waves to generate heat in specific tissues, promoting their regeneration without the need for surgical intervention. Additionally, it stimulates collagen production [15] and improves vascularization [16] and pain [17]. While it is true that RF therapy may have limitations in terms of tissue penetration depth, which may restrict its effectiveness for deeper structures and require several sessions to achieve optimal results, it is considered a very versatile technique, being useful in musculoskeletal injuries [18], wound healing [16], and skin rejuvenation [19]. On the other hand, RF therapy generally has few side effects, with most patients experiencing mild, transient discomfort at the treatment site.

RF devices can be used in various fields of dermatology and esthetic medicine, including the correction of periorbital wrinkles, skin laxity of the lower cheeks and nasolabial and infraorbital folds, expression lines, brow lifting, atrophic and hypertrophic acne scars, as well as other skin rejuvenation procedures [20]. Although its application in the correction of esthetic alterations is broad, its mechanism of action in inflammatory processes, whether acute or chronic, is poorly studied and, therefore, its use in inflammatory skin diseases is still scarce.

Fractional microneedling radiofrequency (FMR) treatments have become one of the most widely used physical therapies due to their low adverse effects and recovery times [21]. FMR showed clinical and histological improvement of rosacea and could be used as an alternative or in combination with other treatment methods [22]. In addition, bipolar radiofrequency has been shown to have a therapeutic effect on other inflammatory skin diseases such as acne [23], hidradenitis suppurativa [24], or seborrheic dermatitis [25].

On the other hand, RF has been proposed as an alternative energy source to induce hyperthermia in nanoparticle (NP) therapies, targeting a specific cell type [26]. Thus, the combination of both techniques could improve RF specificity by concentrating its action on a specific target [27]

CRET therapy uses 448 kHz frequency currents to generate various tissue responses without heating the tissue. Since this therapy has the advantage of being noninvasive, it has been shown to be useful for the treatment of cellulite [28], wrinkle reduction [29], alopecia [30,31], and for the reduction of edema [32]. At the cellular level, previous studies carried out under subthermal conditions have revealed that CRET is capable of inducing the proliferation of hypodermal stem cells [33], fibroblasts, and cutaneous keratinocytes, as well as the migration of fibroblasts [34]. Although there is increasing clinical evidence of the efficacy of this RF on acute and chronic inflammatory pathologies, its effects at the tissue and cellular level have not yet been completely elucidated.

Keratinocytes are the main cell type of the epidermis and are the first receptors of exogenous factors in the skin [2,35]. Both in the inflammatory processes that lead to tissue repair after acute damage and in the repair of chronic injuries, the role of keratinocytes is crucial, since in addition to participating in wound closure through their proliferation and migration, they synthesize multiple cytokines involved in the regulation of wound repair [2,36,37]. Thus, keratinocytes produce cytokines such as interleukins Il-1, -6, -7, -8, -10, -12, -15, -18, and -20, tumor necrosis factor alpha (TNF-α), and interferon-α, β and γ. Receptors activation leads to the activation of proteins such as nuclear factor kappa B (NF-κB) [38], which in turn promotes the expression of target genes, including those of inflammatory cytokines. The production of these cytokines by keratinocytes has multiple consequences for the migration of inflammatory cells and may have systemic effects on the immune system, while also influencing the proliferation, differentiation, and production of cytokines by other keratinocytes [39]. Therefore, an imbalance in the production of these cytokines has been linked to the perpetuation of immunological and inflammatory events described in the pathophysiology of skin pathologies such as lichen planus, psoriasis, epidermolysis bullosa, dermatitis herpetiformis, contact dermatitis, or atopic dermatitis [37,39,40]. In the present study, we investigated the effect of CRET in HaCat on the production of IL-8, MCP-1, RANTES, IL-6, and TNF-α. Additionally, we analyzed the expression and activation of NFκB, MMP, ERK1/2, and the EGF receptor.

Regarding chemokines, IL-8 is a pro-inflammatory cytokine and a neutrophil chemotactic factor, which is commonly produced in the skin after exposure to stimuli such as irritants or contact sensitizers. Moreover, IL-8 levels were found to be increased in immune-mediated blistering diseases such as pemphigus vulgaris [41]. On the other hand, RANTES and MCP-1 are expressed by basal keratinocytes in inflammatory environments [42]. RANTES promotes T cell proliferation and apoptosis and the release of pro-inflammatory cytokines [43]. MCP-1 is produced almost exclusively during the early phase of wound regeneration. It is massively released by wound-edge keratinocytes and macrophages and is involved in the chemotaxis of other cell types such as macrophages and lymphocytes. It may also contribute to endothelial cell locomotion during angiogenesis.

The cytokine IL-6 plays a central role in acute inflammation and is necessary for proper wound resolution. Thus, in response to a wound, keratinocytes promote its early release, which induces the production of pro-inflammatory cytokines by tissue-resident cells such as macrophages, other keratinocytes, endothelial cells, or stromal cells and, like IL-8, induces chemotaxis of wound neutrophils. However, during the inflammatory process, IL-6 is responsible for the activation of the reparative process, since it stimulates the proliferation of keratinocytes [44]. For its part, the cytokine IL-11 is capable of modulating the production of cytokines from dermal and epidermal cells [39] and has been described to minimize tissue damage, since it regulates epidermal cell proliferation and inhibits apoptosis and the production of cytokines from macrophages [45,46].

On the other hand, TNF-α induces cell proliferation, differentiation, apoptosis, and inflammation in tissues. It can act in an autoendocrine manner on keratinocytes and induce the expression of IL-8 [47]. In addition, through the TNF receptor, the activation of NF-κB, a key transcription factor in the inflammatory process, is induced. This activation of NF-κB causes the expression of pro-inflammatory cytokines, chemokines, and adhesion molecules, enhancing and amplifying the immune response of keratinocytes [37]. TNF-α is expressed in keratinocytes after various stimuli and has been observed in injured keratinocytes from patients with epidermotropic cutaneous T cell lymphomas [39].

In keratinocytes, MAP Kinases and the EGFR/ERK pathway participate in proliferation but also actively in the inflammatory process. The binding of growth factors, such as TGF-α or EGF, to the EGF receptor, or their physical or chemical stimulation, activates the Ras/ERK pathway [48]. ERK activation in turn activates transcription factors, including NF-κB as well as the transcription of proteins involved in wound healing such as metalloproteinases (MMPs) [49].

Given the relevance of the above-mentioned molecules in the inflammatory process, the objective of this study was to investigate the effect of RF currents used in CRET therapy on their expression and/or release, as well as keratinocyte proliferation. For this purpose, cell proliferation (XTT assay), cytokine and TNF-α production (ELISA), and expression and activation of NF-κB MMP9, MMP1, ERK1/2, and EGF (immunofluorescence and immunoblot) were analyzed.

## 2. Results

### 2.1. Production of IL-8, MCP-1, RANTES, IL-6, TNF-α, and IL-11

**Figure 1:**
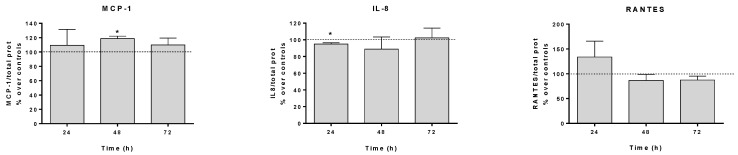

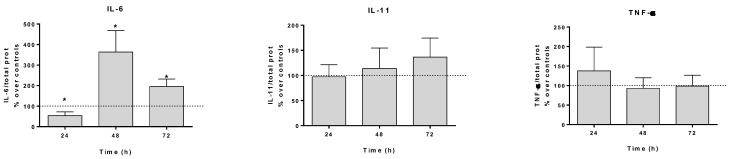
**ELISA.** Content of IL-8, MCP-1, RANTES, IL-6, IL-11, and TNF-α in control or CRET-treated culture medium after 24, 48, or 72 h. Data are means ± SEM normalized to the corresponding controls. The dashed line represents the control group: 100%. Six experimental replicates per time interval and protein. *: 0.05 ≤ *p* < 0.01 Student’s *t* test.

The supernatant of HaCaT cells treated with CRET for 24, 48, or 72 h, and that of their respective controls, was collected and analyzed for cytokine content by ELISA. CRET treatment modified the amount of cytokines produced by HaCaT and released into the culture medium. At 24 and 48 h, the amount of IL-8 decreased in the medium (at 24 h in a statistically significant manner), while it did not change at 72 h compared to the control. IL-6 was also reduced after 24 h of CRET, but at 48 and 72 h of treatment, it increased notably and statistically significantly, compared to the control. MCP-1 and IL-11 showed a tendency to increase compared to the control at all selected time-points, although only MCP-1 levels were significantly increased with respect to the controls after 48 h of CRET. Regarding RANTES, it also showed a tendency to increase in culture media treated with CRET after the first 24 h of treatment. On the contrary, after 48 or 72 h it decreased compared to the control, although none of these results were statistically significant. Finally, a relevant but not statistically significant increase in TNF-α was observed after 24 h of stimulation with CRET, an effect that was lost when the electrical treatments were prolonged until 48 or 72 h (Figure 1).

### 2.2. Proliferation

**Figure 2:**
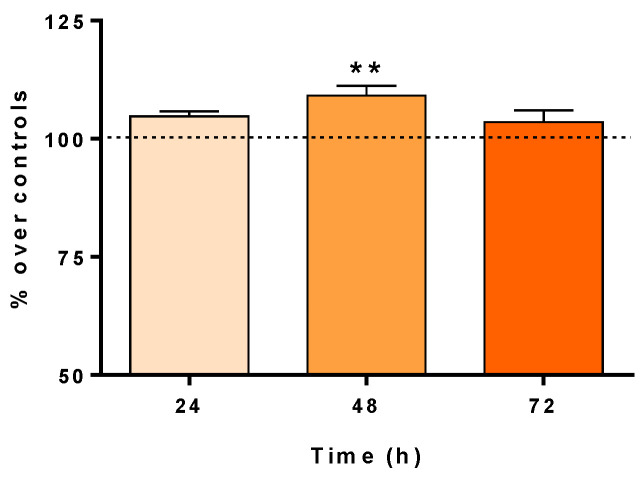
**XTT assay.** Cell proliferation in HaCaT cells treated with CRET after 24, 48, or 72 h. Results are expressed as a percentage with respect to untreated control cells. Data are means ± SEM normalized over the corresponding controls. Dash line represents the control group: 100%. Six experimental replicates per time interval. **: 0.001 ≤ *p* < 0.01. Student’s *t*-test.

After the medium had been collected for cytokine and TNF-α analysis, an XTT assay was performed to determine the effect of CRET on the proliferation of HaCaT keratinocytes. The proliferative rate of HaCaT cells increased slightly after 24, 48, or 72 h of CRET treatment, but the difference was statistically significant with respect to the control only in 48 h treated cultures (Figure 2).

### 2.3. Localization of NF-κB and p-NF-κB

**Figure 3:**
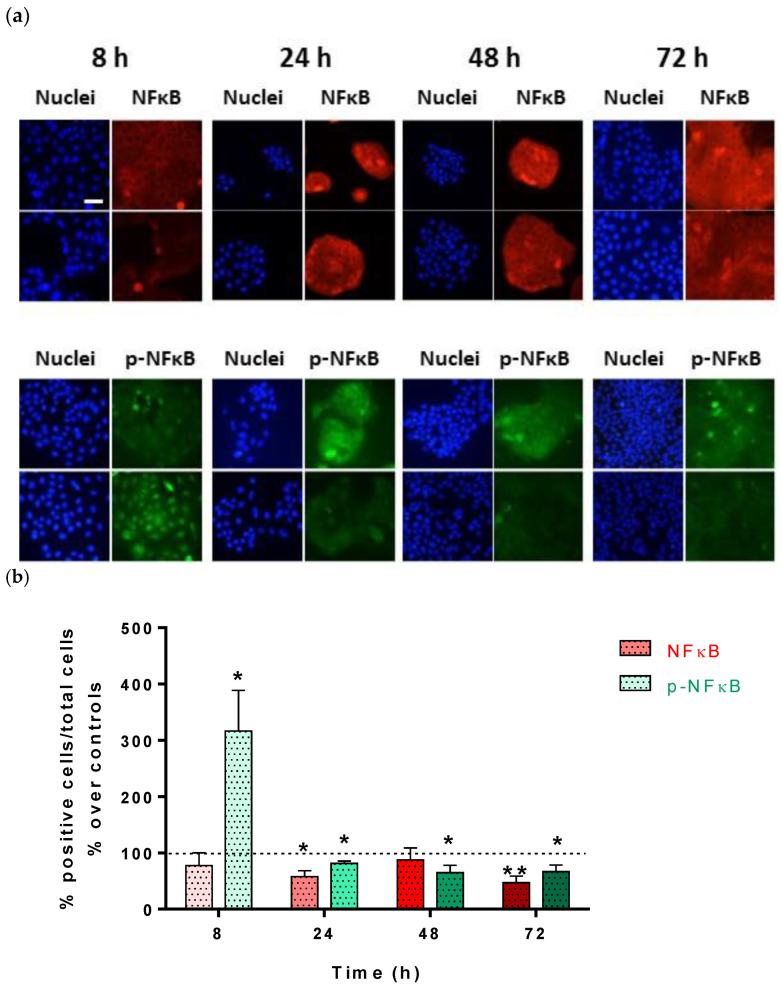
**NF-κB and p- NF-κB immunofluorescence.** NF-κB and p- NF-κB at 8, 24, 48, or 72 h of CRET, or sham treatment. (**a**) Immunofluorescent imaging of NF-κB and p-NF-κB and merged micrographs. Representative micrographs. Red: NF-κB, Green: p-NF-κB, and Blue: cell nuclei. Bar: 50 µm. (**b**) Fluorescence intensity measurement of NF-κB and p-NF-κB proteins per MHC channel. Data are normalized with respect to the corresponding controls. Means ± SEM of the fluorescence intensity/total nuclei of at least three experimental repeats per protein and time interval. **: 0.001 ≤ *p* < 0.01; *: 0.05 ≤ *p* < 0.01. Dash line represents the control group: 100%.

To determine the localization of NF-κB and its activated form (p-NF-κB), this transcription factor was analyzed by fluorescence microscopy. Translocation of NF-ΚB to the nucleus converts it into a transcription factor (p-NF-κB) that promotes the synthesis of various proteins, which in turn activate pathways involved in proliferation and inflammation. Regarding its localization, only the 8 h samples showed a nuclear localization of p-NF-κB, which was very abundant in the CRET-treated group compared to their controls. At the rest of the analyzed moments (24, 48, or 72 h), the localization of p-NF-κB was scarce and mainly cytoplasmic in both treated and untreated cells (Figure 3a).

When the labeling of both proteins was assessed, NF-κB had reduced expression at all times analyzed, being statistically significant after 24 and 72 h of CRET treatments. The activated protein (p-NF-κB) increased very notably at 8 h of CRET treatment, while it decreased slightly, but in a sustained and statistically significant manner at 24, 48, or 72 h, compared to the control (Figure 3b).

### 2.4. Expression of EGFR, p-EGFR, ERK1/2, p-ERK1/2, MMP1, and MMP9

**Figure 4:**
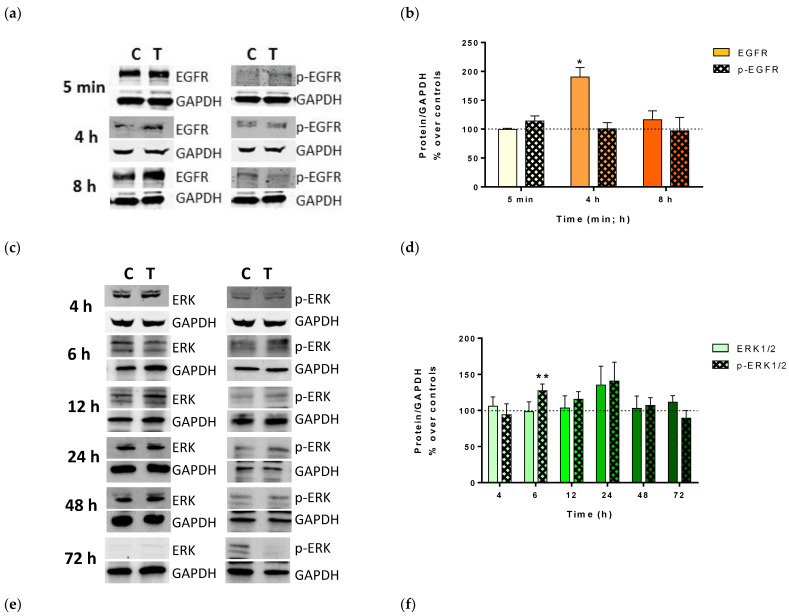

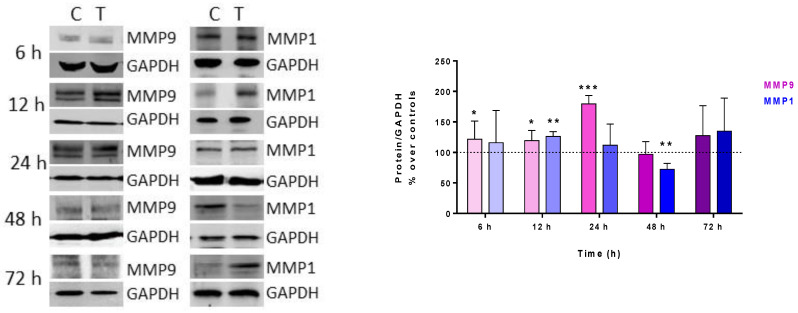
**EGFR, p-EGFR, ERK1/2, p-ERK1/2, MMP9, MMP1, and GAPDH immunoblots**. (**a**) Representative EGRF and p-EGFR blots (30 µg protein/lane) at 5 min, 4 h, or 8 h of CRET treatment or control. (**b**) Assessment of EGFR and p-EGFR expression. Densitometry of immunoblots. (**c**) Representative ERK and p-ERK blots (30 µg protein/lane) at 4, 6, 12, 24, 48, or 72 h of CRET treatment or control. (**d**) Assessment of ERK1/2 and p-ERK1/2 expression. Densitometry of immunoblots. (**e**) Representative MMP9 and MMP1 blots at 6, 12, 24, 48, or 72 h of CRET treatment or control. (**f**) Assessment of MMP9 and MMP1 expression. Densitometry of immunoblots. For figures (**a**,**c**,**e**): 30 µg protein/lane. C: Control. T: CRET treatment. GAPDH was used as loading control. For figures (**b**,**d**,**f**): means ± SD of the protein/GAPDH ratios of at least four experimental repeats per protein and time interval. ***: 0.0001 ≤ *p* < 0.00; **: 0.001 ≤ *p* < 0.01; *: 0.05 ≤ *p* < 0.01. Student’s *t*-test. Dotted line: control group (100%); solid bars: inactivated proteins (EGFR or ERK1/2); dotted bars: activated proteins (p-EGFR or p-ERK1/2).

EGFR was analyzed immediately after the end of each pulse, because it is a membrane receptor, and therefore the first protein to receive the electrical stimulus. Therefore, it is reasonable to assume that any effect that may occur on it should be relatively rapid and should be detected preferably during the first stimulations. Thus, EGFR was analyzed immediately after the first (5 min), second (4 h), or third pulse of CRET (8 h). The immunoblot results showed a notable and statistically significant increase in its expression after 4 h of CRET stimulation, although no changes in its activation were detected at any of the times analyzed (Figure 4a,b).

Regarding ERK1/2, its expression in CRET-treated cultures did not vary with respect to the control at any of the moments analyzed (4, 6, 12, 24, 48, or 72 h). However, after 6 h of electrical treatment, its active form (p-ERK1/2) increased significantly with respect to the control in CRET-treated cultures. This increase in p-ERK1/2 was maintained at 12 and 24 h but was not statistically significant with respect to the control. At 72 h, the activation of ERK1/2 in CRET-treated cultures was lower than in controls, but the difference was not statistically significant (Figure 4c,d).

Analysis of MMPs revealed a statistically significant increase in MMP9 expression over the control at 6, 12, and 24 h of CRET treatment. Regarding MMP1, its expression increased at 12P-1/ h but decreased at 48 h compared to the control (Figure 4e,f).

## 3. Discussion

It has been described that stimulation with electromagnetic fields and electric currents can induce wound healing through modulations in the inflammatory process, cell proliferation, migration, and wound healing [50,51,52,53]. In the present study, the effect of RF currents used in CRET therapy was analyzed on molecules that regulate the inflammatory and regenerative process in the skin, such as the EGF membrane receptor, the MAP Kinase ERK1/2, the transcription factor NF-κB, MMP1 and MMP9, and the expression of cytokines IL-6, IL-11, RANTES, IL-8, MCP-1, and TNF-α.

CRET treatment induced EGFR overexpression, which would activate ERK1/2 through the Ras/ERK pathway. It has been described that RF exposure is capable of stimulating this pathway through the generation of free radicals (ROS) in the membrane, which would activate the EGF receptor [54]. It is also known that exposure of HaCaT to direct current electric fields induces an activation of this signaling pathway that can generate, at least partially, migration, proliferation, and cytokine secretion [55].

However, if p-ERK activation is prolonged, the Ras/ERK1/2 pathway ends up being inhibited [56,57]. In this study, a sustained activation of ERK1/2 was found during the first 48 h of CRET treatment. When treatment was prolonged beyond 48 h, ERK1/2 activation was not detected, which would indicate an inhibition of the pathway. In terms of proliferation, this initial activation would be responsible for the increase in keratinocytes detected (see Figure 2).

In inflammation, the activation of EGFR and the Ras/ERK1/2 pathway activates the transcription factor NF-κB [49]. In our cultures, CRET induced a very relevant increase in the activation of NF-κB in the first 8 h of treatment, but at 24 h and successive time intervals, NF-κB was deactivated and its expression decreased. It is likely that this transient increase in the expression of NF-κB induced by CRET is caused by the activation of the ERGR/ERK1/2 pathway and its subsequent inhibition. Exposure to ELF-EMF can also modulate the production of chemokines by blocking the NF-kB signaling pathway [58], thus inhibiting inflammatory processes. Other physical therapies such as 1 MHz ultrasound also cause the transient activation of NF-κB in HaCat [59].

Although other studies have shown that low-frequency subthermal electromagnetic fields reduce MCP-1 [58], CRET radiofrequency increased its expression in HaCat. This could be due to the electrical and electromagnetic differences of both physical agents (448 kHz versus 50 Hz). On the other hand, it is known that the production of MCP-1 by keratinocytes attracts monocytes, lymphocytes, and mast cells. In particular, mast cells produce IL-4, an anti-inflammatory cytokine [41]. However, the effect of CRET on the expression of IL-4 in mast cells, and its interaction with keratinocytes or other cell types, has not been studied.

Regarding IL-6, an increase in its production and secretion into the culture media was observed in our experiments. As in MCP-1, CRET could promote the increase in IL-6 secretion through the NF-κB signaling pathway. NF-κB activation and IL-6 upregulation were also detected in keratinocytes exposed to mechanical stress [59] or UV irradiation [60]. IL-6 is known to be a pleiotropic cytokine that coordinates a large number of pro-inflammatory and anti-inflammatory functions, depending on the circumstances [2]. IL-6 modulates the immune response, differentiation and promotes keratinocyte proliferation [61], so its sustained overexpression due to CRET stimulation could contribute to the proliferative effect observed in this study and in previous investigations [34].

Several studies have shown that many inflammatory skin diseases display an abnormal cytokine profile. Thus, keratinocytes from patients with atopic dermatitis present elevated mitosis and continuous production of pro-inflammatory factors with an abnormal release of cytokines such as IL-1, IL-6, TNF-α, MCP-1, RANTES, among others. Keratinocytes from chronic inflammatory pathologies such as psoriasis have intrinsic defects that cause exaggerated production of chemokines such as IL-8, MVCp-1, or CXCL10 and alterations in the production of IL-6, while in contact dermatitis keratinocytes overexpress IL-6, RANTES, IL-8, MCP-1, IL-1α, IL-1β, IL18, and TNF-α [37,62,63]. Furthermore, IL-8 is overexpressed in human chronic wounds [64]. The results of this study showed that, compared to controls, CRET induces a decrease in the production of IL-8 and RANTES. This decrease in IL-8 and RANTES could contribute to a possible anti-inflammatory effect in this type of skin pathology, induced by electrical stimulation. Similarly, stimulation with other types of physical therapy based on electromagnetic fields has revealed anti-inflammatory effects due to its ability to reduce pro-inflammatory chemokines [58], increase the phagocytic activity of macrophages, and induce a reduction in the activity of the enzyme Nitric Oxide synthase, which promotes inflammation [65].

Furthermore, in CRET-treated keratinocytes, IL-11 production and release increased slightly but progressively over time. It has been speculated that IL-11 may play an important role in the resolution of skin inflammation in patients with psoriasis [66] and plays an important role in tissue remodeling [45]. It is conceivable that a potential increase in IL-11 production induced by CRET could contribute to wound healing and/or to the resolution of inflammatory pathologies.

In fact, p-NF-κB also participates in wound healing processes through its activity on MMPs. These MMPs are essential for tissue regeneration and wound healing because they are involved in the degradation and regeneration of the extracellular matrix (ECM) [62,67]. MMP9 is produced by migrating keratinocytes to degrade collagen and laminin from the basement membrane, allowing their migration until wound resolution. MMP9 is also associated with ECM remodeling after wound closure and its overexpression is associated with chronic wounds and other skin disorders such as epidermolysis bullosa or cicatricial pemphigoid [68]. In CRET-treated keratinocytes, the initial increase in NF-κB expression would induce the early expression of MMP1 and MMP9 observed. In a tissue, this would induce the degradation of the ECM, allowing the proliferation and/or migration of keratinocytes and other surrounding cells involved in tissue regeneration and inflammation such as fibroblasts, endothelial cells, mast cells, or immune cells. Thus, the effect of CRET could also be exerted on tissue regeneration through ECM remodeling as previously proposed by Meyer et al. [15] and in previous studies by our group [34]. Other studies have also shown that exposure to ELF-EMF could be useful in wound repair because it is able to promote keratinocyte proliferation/migration by modulating MMP9 expression through the Akt/ERK pathway [65]. The subsequent inactivation of the MAPK-NF-Kb pathway induced by CRET would cause a reduction in MMP1, thus promoting wound healing in damaged tissue.

TNF-α is a potent pro-inflammatory cytokine and its regulation in the skin is complex, as its effects may vary depending on the specific context and pathological scenarios. Thus, TNF-α itself can stimulate the release of anti-inflammatory factors such as IL-10, endogenous corticosteroids, and prostanoids, which are able to downregulate and counteract its expression, contributing to the control of the progress of inflammation and also inhibiting its extension and duration. Its deregulation can cause hyperproliferation of keratinocytes, leading to psoriasis or inducing cutaneous lupus erythematosus [68]. TNF-α, acting together with NF-κB, modulates the expression of the matrix metalloproteinase (MMP) gene and induces the production of MMP9 [69]. In our cultures, TNF-α was increased in the culture medium at 24 h. Therefore, this increase will also contribute to the higher expression of MMP9 observed after electrical treatment. Taken together, these effects induced by CRET in keratinocytes, if they occur in an injured tissue, could lead to better control of its inflammation.

## 4. Materials and Methods

### 4.1. Cell Culture

Human epidermal keratinocytes HaCaT (CLS Cell Lines Service, 300493, Heidelberg, Germany) were maintained in a medium composed of high-glucose D-MEM (Biowhittaker, Lonza, Verviers, Belgium) supplemented with 10% inactivated fetal bovine serum (Gibco Waltham, MA USA 02451), 1% glutamine, and 1% penicillin–streptomycin (Gibco). Cells were incubated at 37 °C in a humidified atmosphere containing 5% CO_2_ and subcultured once a week in an F-75 flask. The medium was changed every 3 days.

For the experiments, cells were subcultured once a week and plated on the bottom of 60 mm Petri dishes (Nunc, Roskilde, Denmark), except for immunofluorescence assays, in which the cells were seeded on glass coverslips placed on the bottom of the plates. Cells were periodically tested for mycoplasma.

### 4.2. Electrical Treatment

Three or four days after seeding, depending on the experiment, ad hoc sterile stainless steel electrode pairs designed for in vitro stimulation were inserted into all Petri dishes and connected in series [70]. Cultures intended for electrical stimulation (CRET-treated) were connected to a CRET device (model INDIBA Deep Care ELITE NS, INDIBA^®^, Barcelona, Spain). The remaining cultures (control) were incubated in the same CO_2_ incubator but not connected to the CRET device. A 448 kHz sinusoidal current was applied at a subthermal current density of 100 µA/mm^2^. The intermittent stimulation pattern consisted of 5 min pulses separated by 4 h interpulse intervals. This current pattern and density were selected because they have previously demonstrated significant effects on keratinocyte, fibroblast, and ADSC cultures [33,34]. Depending on the biomarker analyzed, treatments lasted 5 min or 4, 6, 8, 12, 24, 48, or 72 h.

### 4.3. ELISA

Cytokines MCP-1, RANTES, IL-8, IL-11, IL-6, and TNF-α were analyzed by Enzyme-Linked ImmunoSorbent Assay (ELISA). HaCat cells were seeded at a density of 4500 cells/cm^2^ and incubated for 4 days, at which time stimulation was started. At the end of electrical treatment or sham treatment (24, 48, or 72 h), the medium was collected from the HaCat cultures and processed to determine the concentration of chemokines following the protocols established in the following kits: the Human CCL3/MIP-1 alpha Quantikine ELISA Kit (Catalog nº: DMA00), Human CCL2/MCP-1 Quantikine ELISA Kit (Catalog nº: DCP00), Human CCL5/RANTES Quantikine ELISA Kit (Catalog nº: DRN00B), and Human IL-8/CXCL8 Quantikine ELISA Kit (Catalog nº: D8000C) all from RyDBiosistems (RyDBiosistems; UK). IL-11 (Catalog nº: AB189569), IL-6 ((Catalog nº: AB178013), and TNF-α (Catalog nº: AB181421) were all procured from Abcam (Abcam; Cambridge, UK). Because CRET treatments exert an effect on HaCat proliferation, ELISA data were normalized to the total protein content of the collected media. Total protein concentration was determined using a Pierce BCA protein assay (Catalog nº 23225, Thermo Fisher Scientific, Inc; Waltham, MA, USA).

### 4.4. Cell Proliferation

HaCat proliferation was tested using the Cell Proliferation Kit II (XTT) (product number 11465015001; Roche, Switzerland). HaCaT cultures were seeded at densities of 4500 cells/cm^2^ and incubated for 3 days. After 24, 48, or 72 h of treatment with CRET or sham treatment, cells were incubated for 3 h with XTT tetrazolium salt in an atmosphere of 37 °C and 6% CO_2_, as recommended by the manufacturer. Metabolically active cells reduced XTT into colored formazan compounds which were quantified with a microplate reader (TECAN, Mannedorf, Switzerland) at a wavelength of 492 nm.

### 4.5. Inmunoblot

HaCaT cells were plated at 6800 cell/cm^2^ density and incubated for 4 days. RF or sham-treated cells were lysed for protein extraction at 5 min or 4, 6, 8, 12, 24, 48, or 72 h from the treatment onset. The immunoblot procedure has been described in detail previously [70]. Briefly, 30 μg protein of lysed protein was separated in 10% sodium dodecyl sulphate–polyacrylamide gel and electrophoretically transferred to nitrocellulose membrane (Amersham, Buckinghamshire, UK). The membranes were incubated at 4 °C overnight in anti-MMP9 rabbit monoclonal antibody (1:1000, Abcam, UK), anti-MMP1 rabbit monoclonal antibody (1:1000; Abcam), anti-p-ERK1/2 rabbit polyclonal antibody (1:1000; Thermo Fisher Scientific), anti-ERK1/2 polyclonal antibody (1:1000; Cell Signaling, Danvers, MA, USA), anti-EGFR mouse monoclonal antibody (1:1000 Thermo Fisher Scientific), and anti-p-EGFR rabbit antibody (1:1000, Cell Signaling). Anti-GAPDH (1:1000, Santa Cruz Biotechnology, Dallas, TX, USA) was used as a loading control. The membranes were incubated for one hour at room temperature with anti-rabbit IgG conjugated to IRdye 800 CW (1:10,000, LI-COR Biosciences, Lincoln, NE, USA) and with anti-mouse IgG conjugated to IRdye 680 LT (1:15,000, LI-COR Biosciences). Then, the membranes were scanned with a LI-COR Odyssey scanner (LI-COR Biosciences). The obtained bands were densitometry evaluated (PDI Quantity One 4.5.2 software, BioRad, Hercules, CA, USA). At least 3 experimental replicates were conducted per protein and cell type. All values were normalized over the loading control.

### 4.6. Immunofluorescence

For immunofluorescence, cells were seeded at a density of 13,600 cells/cm^2^ and incubated for 4 days. After 8, 24, 48, or 72 h of CRET or sham treatment, the samples were fixed with 4% paraformaldehyde at 4 °C for 20 min. Cultures were incubated overnight at 4 °C with anti-NF-κB p65 (F-6) mouse monoclonal antibody (1:100, Santa Cruz Biotechnology) and anti-p-NF-κB p65 (Ser536) (93H1) rabbit mAb antibody (1:1000, Cell Signalling). Afterwards, the samples were fluorescence stained with Alexa Fluor^®^ 488 goat anti-rabbit IgG (1:500, Invitrogen, Thermo Fisher Scientific) or Alexa Fluor^®^ 568 conjugate goat anti-mouse IgG (1:500, Invitrogen, Carlsbad, CA, USA) for 1 h at room temperature, and the cell nuclei were counterstrained and mounted with ProLong-DAPI (Invitrogen). Images of the cultures were acquired using an inverted fluorescence microscope (Nikon Eclipse Ts2R) coupled to a digital camera (Nikon DS-Ri2) and analyzed with image analysis software (NIS-Elements, (version 4.40, NIKON, Melville, NY, USA). To assess staining intensity, RGB fluorescence thresholds (MCH mode) were set prior to analysis and applied to all images. Using the NIS-Elements program, all cells with a fluorescence value greater than or equal to the previously set value were counted as positive. The number of positive cells was normalized to the total number of cells counted per field.

### 4.7. Statistical Analysis

At least three independent replicates were performed per experiment. To assess statistically significant differences between the CRET-treated and the control groups, the unpaired Student *t* test was applied. Statistical analysis was performed using GraphPad Prism 6.01 software (GraphPad Software, San Diego, CA, USA). Differences *p* < 0.05 were considered statistically significant.

## 5. Conclusions

This study provides information on the effect of CRET on the inflammatory process triggered by keratinocytes, as well as on the signaling molecules that modulate this process. Thus, the results indicate that CRET modulates the expression of several cytokines, TNF-α and NF-κB, through the EGFR/ERK1/2 pathway. Since an excessive or prolonged inflammatory response induces poor and delayed healing, any therapeutic tool that can regulate the pro-inflammatory process may have a potentially relevant clinical application. Therefore, although these results come from an in vitro study, which could be a limitation for their direct extrapolation to patients, they provide robust evidence that these CRET currents could be a good therapeutic option for inflammatory skin pathologies and could help optimize the time of conventional treatments. Furthermore, previous studies have shown that CRET promotes the proliferation and migration of fibroblasts and keratinocytes, so, in addition to an anti-inflammatory action, CRET would exert a regenerative action during the inflammatory and proliferative phases of wound healing. Although experimental evidence and clinical cases are increasingly numerous and reveal the usefulness of this therapy and other similar physical therapies in inflammatory processes, clinical trials are required to characterize the possible applications of these therapies in inflammatory skin pathologies. This study provides a better understanding of a valuable field of research for the development of future therapeutic tools that accelerate skin regeneration and wound closure, in a less invasive way and with fewer side effects than those currently used.

## Data Availability

The data presented in this study are available on request from the corresponding author. The data are not publicly available due to privacy restrictions.

## Abbreviations

**ADSC**	Adipose-derived stem cell
**AKT**	Protein kinase B
**CCL**	C-C motif ligand
**CRET**	Capacitive resistive electric transfer
**ECM**	Extracellular matrix
**EGF**	Epidermal growth factor
**EGFR**	Epidermal growth factor receptor
**ELISA**	Enzyme-linked immunoassay
**ERK1/2**	Extracellular signal-regulated protein kinases 1 and 2
**FMR**	Fractional microneedling radiofrequency
**GAPDH**	Glyceraldehyde-3-phosphate dehydrogenase
**IgG**	Immunoglobulin G
**IL**	Interleukin
**IPL**	Intense pulsed light
**kHz**	kilohertz
**MAPK**	Mitogen-activated protein kinase
**MCP-1**	Monocyte chemoattractant protein-1
**MMP**	Matrix metalloproteinases
**NF-κB**	Nuclear factor kappa-light-chain-enhancer of activated B cells
**NP**	Nanoparticles
**PDL**	Pulsed dye laser
**RANTES**	Regulated on activation, normal Tcell expressed and secreted
**Ras**	Rat sarcoma virus
**RF**	Radiofrequency
**TNF**	Tumor necrosis factor
